# Red/Orange Autofluorescence in Selected Candida Strains Exposed to 405 nm Laser Light

**DOI:** 10.3390/dj12030048

**Published:** 2024-02-26

**Authors:** Rafał Wiench, Dariusz Paliga, Anna Mertas, Elżbieta Bobela, Anna Kuśka-Kiełbratowska, Sonia Bordin-Aykroyd, Aleksandra Kawczyk-Krupka, Kinga Grzech-Leśniak, Monika Lukomska-Szymanska, Edward Lynch, Dariusz Skaba

**Affiliations:** 1 Department of Periodontal Diseases and Oral Mucosa Diseases, Faculty of Medical Sciences in Zabrze, Medical University of Silesia, 40-055 Katowice, Poland; rwiench@sum.edu.pl (R.W.); anna.kuska-kielbratowska@sum.edu.pl (A.K.-K.); dskaba@sum.edu.pl (D.S.); 2 Dental Office Reanata and Dariusz Paliga, Aleja Niepodległości 3/lok 2, 35-303 Rzeszów, Poland; dpaliga@data.pl; 3 Department of Microbiology and Immunology, Faculty of Medical Sciences in Zabrze, Medical University of Silesia, 40-055 Katowice, Poland; amertas@sum.edu.pl (A.M.); ebobela@sum.edu.pl (E.B.); 4 Photomedicine, Leicester School of Pharmacy, De Montfort University, The Gateway, Leicester LE1 9BH, UK; dr.sonia@visagelaserinstitute.com (S.B.-A.); p2628527@my365.dmu.ac.uk (E.L.); 5 Department of Internal Diseases, Angiology and Physical Medicine, Center for Laser Diagnostics and Therapy, Medical University of Silesia in Katowice, 41-902 Bytom, Poland; akawczyk@sum.edu.pl; 6 Laser Laboratory, Dental Surgery Department, Wroclaw Medical University, 50-425 Wroclaw, Poland; 7 Department of Periodontics, School of Dentistry, Virginia Commonwealth University, Richmond, VA 23284, USA; 8 Department of General Dentistry, Medical University of Lodz, 251 Pomorska St., 92-213 Lodz, Poland; monika.lukomska-szymanska@umed.lodz.pl

**Keywords:** yeast, autofluorescence, oral candidiasis, porphyrins, protoporphyrin, fluorophore, diode laser

## Abstract

Background: *Candida albicans* and similar species are significant pathogens in immunocompromised and hospitalized individuals, known for mucosal colonization and bloodstream/organ invasion. Many pathogenic *fungi,* including these species, exhibit autofluorescence (R/OF) under specific light conditions, a feature crucial for their detection. Aim: We investigated the use of a 405 nm diode laser for the direct observation of red/orange autofluorescence of *Candida* spp., common in the oral cavity, exploring its potential in health screenings. Methods: This study utilized cultures of *Candida* spp. on Sabouraud dextrose agar with Qdot 655 and 685 for fluorescence benchmarking, illuminated using a 405 nm diode laser (continuous wave, power 250 mW, 0.0425 J/cm² fluence, 0.0014 W/cm² power density). Images were captured using a yellow-filter camera at set intervals (48 to 144 h). Visual and computational analyses evaluated the R/OF in terms of presence, intensity, coloration, and intra-colony variation. Results: Most *Candida strains* displayed red/orange autofluorescence at all observation times, characterized by varied coloration and intra-colony distribution. Initially, there was an increase in R/OF intensity, which then stabilized in the later stages of observation. Conclusions: The majority of the *Candida strains* tested are capable of emitting R/OF under 405 nm laser light. This finding opens up new possibilities for integrating R/OF detection into routine dental screenings for *Candida* spp.

## 1. Introduction

Candidiasis frequently affects human skin, oral and esophageal areas, the gastrointestinal tract, and the vagina. While it primarily occurs in those with compromised immune systems or other debilitations, *Candida albicans,* the primary causative agent, exhibits multiple virulence factors that aid in its pathogenicity [1,2,3,4].

The prevalence of candidiasis, infections caused by *Candida* species, has significantly increased in recent decades. This rise is attributed to factors such as the AIDS and COVID-19 epidemics, the world’s aging population, the higher number of immunocompromised individuals, and the common use of contaminated medical devices [5,6,7]. This holds significant clinical relevance, given that *Candida albicans* is present in 40% of healthy individuals as a typically benign organism in the oral cavity [8]. However, in scenarios where patient immunocompetence is compromised, *Candida albicans* can lead to not only oral candidiasis but also invasive forms of the disease. Additionally, *Candida albicans* poses a risk for oral squamous cell carcinoma due to its capacity to stimulate the production of carcinogenic substances, including endogenous nitrosamines. Specifically, *Candida albicans* can transform nitrite into nitrosamines, concurrently producing acetaldehyde, which plays a carcinogenic role in the oral cavity [9]. 

While *Candida albicans* remains the primary cause, non-*albicans Candida* species like *Candida glabrata, Candida tropicalis,* and *Candida parapsilosis* are increasingly being recognized as human pathogens [10]. 

Autofluorescence shows promise as a diagnostic tool capable of detecting biofilms and cellular alterations by providing both morphological and biochemical insights [11]. The emission wavelengths vary by tissue type and the number of fluorophores present [12,13,14]. The light-absorbing molecules are known as photo-acceptors (chromophore, fluorophore). Endogenous fluorophores, which can be excited to emit autofluorescence intrinsically within cells and tissues, include oxidoreductase coenzymes such as flavin adenine dinucleotide (FAD), nicotinamide adenine dinucleotide (NAD), and a reduced form of nicotinamide adenine dinucleotide (NADH); amino acids and proteins (tryptophan, phenylalanine, tyrosine, elastin, keratin, collagen); pyridine derivatives (pyridoxine, pyridoxyl phosphate, pyridoxylic acid); heme synthesis intermediates (porphyrin); and lipophorin metabolism products. In contrast, light-absorbing chromophores such as melanin and hemoglobin do not fluoresce and can reduce tissue fluorescence [15].

The autofluorescence characteristics of the oral mucosa are contingent upon the specific anatomical location and the nature of any concurrent lesions. In normal mucosa, autofluorescence within the ultraviolet (UV) and visible regions of the spectrum emits a pale-green fluorescence. In contrast to healthy tissue, pathological tissue displays variable levels of fluorescence visualization loss (FVL), characterized by a dark brown to black mucosal area or by red/orange light emission. This phenomenon can serve as a non-specific indicator of structural irregularities or congestion within the tissue, potentially signifying heightened metabolic activity such as increased cell division, which may contribute to the development of potentially malignant or neoplastic conditions [16,17]. On the other hand, it may also indicate the presence of inflammatory and infective processes [15]. 

All the above characteristics collectively influence the manifestation of fluorescence loss, with its extent correlating to the level of tissue damage. McAlpine et al. delineated four distinct patterns of mucosal autofluorescence:

1. Normal healthy tissue emitting a pale green fluorescence.
2. Loss of fluorescence (LOF), distinguished by a dark brown to black mucosal area and typically associated with dysplastic and/or neoplastic tissues.
3. Gained fluorescence (GF), commonly observed in hyperkeratotic lesions like leukoplakia.
4. Porphyrin fluorescence (PF), characterized by a red/orange light emission at a peak of 636 nm, attributed to porphyrins generated by microorganisms [18,19,20,21].

Biofilms are particularly prevalent on the dorsal surface of the tongue, as well as the hard and soft palate, gums, and tooth surfaces. In these regions, a R/OF often overlays the green autofluorescence pattern of the mucosa and enamel itself [20,21,22]. 

*Candida* species commonly found in biofilms are suspected to contribute to porphyrin fluorescence (PF) due to the confirmed presence of endogenous porphyrins in their membranes [23,24,25,26].

To date, conclusive evidence remains elusive regarding the sufficiency of porphyrins within yeast cells for their detection via autofluorescence-inducing techniques. Consequently, a research hypothesis posits that *Candida* spp. might be implicated in the observable R/OF in tests. 

Aim: Given the increased incidence and common presence of *Candida* spp. in the oral biofilm and its potential pathogenicity, especially in immunocompromised individuals, this study seeks to determine whether various *Candida* strains commonly found in the oral cavity can be detected via red/orange autofluorescence (R/OF) by direct observation under 405 nm diode laser illumination. This approach could serve as a non-invasive, chair-side, routine-screening diagnostic tool for confirming the presence of certain *Candida* spp. in the oral cavity, which could be the basis for performing a clear objective detection by swab, smear, or oral rinse, followed by a specific microbial culture, as a starting point for the effective care of the patient, thereby significantly enhancing the early detection, prevention, and/or treatment of *Candida*-related conditions including candidiasis and oral squamous cell carcinoma.

## 2. Materials and Methods

### 2.1. Fungal Strains

This study involved reference strains of *Candida* species obtained from the American Type Culture Collection (ATCC, Manassas, VA, USA), including *C. albicans* ATCC 60193, *C. albicans* ATCC 10231, *C. glabrata* ATCC 66032, *C. krusei* ATCC 14243, and *C. parapsilosis* ATCC 20019. These strains, part of the strain bank at the Department of Microbiology and Immunology, Faculty of Medical Sciences in Zabrze, Medical University of Silesia in Katowice, Poland, were chosen for their prevalence as etiological agents of oral candidiasis [27]. They were preserved at −80 °C in tryptic soy broth with glycerol.

### 2.2. Inoculum Preparation

Each *Candida* strain was individually cultured on Sabouraud dextrose agar (SDA) with 4% glucose (BTL, Łódź, Poland) and incubated aerobically at 37 °C for 24 h. Post incubation, yeast colonies were aseptically transferred from the agar into a sterile 0.9% NaCl solution. The viable cell concentration in the suspension was measured using a Densimat spectrophotometer (bioMerieux, Marcy I’Etoile, France) at a 950 nm wavelength. The optical density of the samples was aligned with a McFarland standard of 0.5, equating to approximately 1–5 × 10^6^ viable cells per milliliter.

### 2.3. Study Groups and Culture Conditions

#### 2.3.1. Study Groups

**Figure 1:**
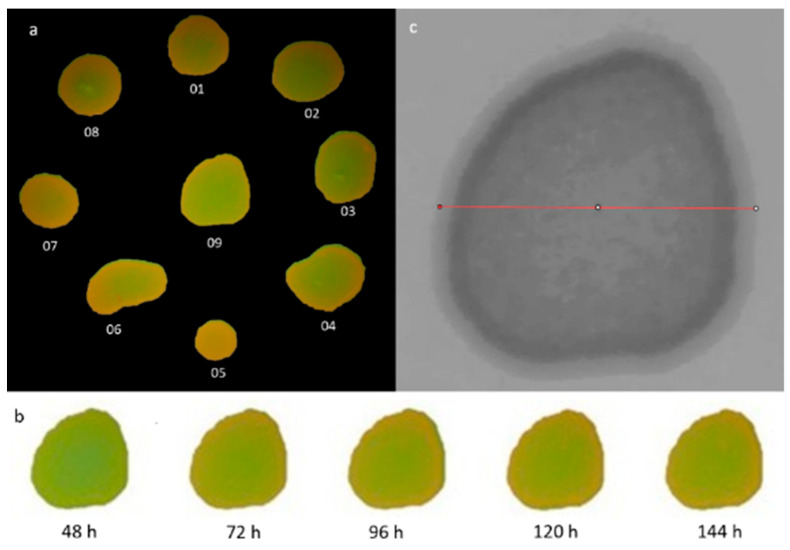
(**a**). The colony identification methodology employed on the experimental plate, featuring *Candida albicans* strain ATCC 60193. (**b**). A representation of colony number 09 from *Candida albicans* ATCC 60193, prepared for analysis within the GIMP software version 2.99.16, corresponding to specific observation periods. (**c**). The measured sample diameter for colony number 09 of *Candida albicans* ATCC 60193, prepared for analysis using the ImageJ-Fiji program, version 1.53. Redline—a bisecting diameter segment that spanned the entire colony area parallel to the image baseline.

Aliquots (10 μL) of fresh *Candida* suspensions were precisely inoculated onto SDA plates in a predefined circular pattern using a sterile pipette. Each plate featured eight equidistant colonies around the perimeter and one central colony, following a specific template (Figure 1a). Five such plates were prepared, each dedicated to a different test strain of *Candida.*

**Figure 2:**
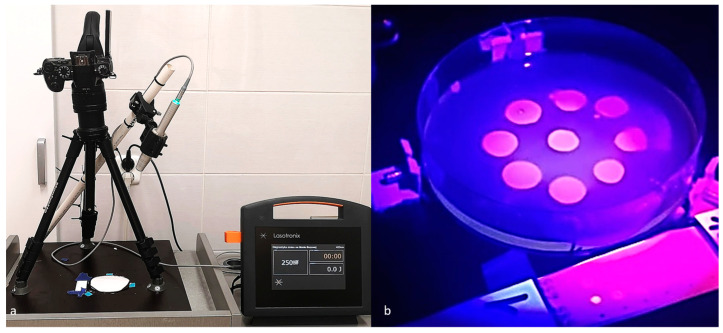
(**a**) A laboratory workspace prepared for experimentation. (**b**) An exemplary test plate and control group quantum dots created under the illumination of a 405 nm laser without the inclusion of the yellow filter utilized in the study.

The plates were incubated aerobically at 37 °C for 48 h. After incubation, photographic images of the plates were acquired using a DMC-G80 Lumix Panasonic Corporation camera (Osaka, Japan) equipped with a Lumix Panasonic H-FS 2060, 12-60 micro-HD lens (Tokyo, Japan). These images were taken under white light illumination and using fixed camera settings from a standardized distance of 34 cm. To ensure the plates remained in a constant position during image capture, three pins were affixed to their external walls, securely stabilizing their placement within a holder affixed to the surface (Figure 2a,b).

#### 2.3.2. Control Group

To evaluate the uniformity of the images and facilitate color comparisons, a control group was introduced. This control group consisted of a paper strip positioned adjacent to the test plate, featuring two rows of dots (Figure 2a,b). The first vertical row of dots, closer to the agar plate, included Qdot TM 685 goat F (ab) 2 anti-mouse IgG conjugate (H + L) solutions provided by Thermo Fisher Scientific (Warsaw, Poland). The lowermost dot contained 1 mL of a 1 µM quantum dot solution, and the four dots above it represented consecutive ten-fold dilutions of the solution in saline. 

The second vertical row (further from the agar plate) contained 1mL of a 1 µM quantum dot solution, Qdot TM 655 goat F (ab) 2 anti-mouse IgG conjugate (H + L) (Thermo Fisher Scientific (Warsaw, Poland), (lowest dot) and their 4 consecutive 10-fold dilutions in saline (dots above it).

The Qdot 655 and 685 probes utilized in this study consist of fluorescent semiconductor nanocrystals that serve as robust orange and red fluorescent labels, exhibiting exceptional brightness and photostability ideal for imaging applications.

An additional control consisting of a consistent area of SDA exhibiting native green autofluorescence was utilized for green color comparison. The dimensions of the control region analyzed corresponded to the size of the lowest concentration orange quantum dot sample.

#### 2.3.3. Light Source

The autofluorescence of the *Candida* colonies was induced using a 405 nm continuous-wave diode laser (Smart^M^Pro, Lasotronix, Piaseczno, Poland) with a rated output power of 250 mW. The laser was equipped with a 12 mm diameter convex glass applicator tip, providing a 1.0 cm^2^ irradiation area with a Gaussian beam profile. Samples were irradiated for 30 s using proprietary software parameters optimized for oral mucosa analysis. The laser handpiece was mounted at a fixed distance of 34 cm above the plates and angled at 65 degrees to the surface in a light-excluded room. Based on the beam area of 176.6 cm^2^, at this geometry, the calculated fluence and power density at the sample plane were 0.0425 J/cm^2^ and 0.0014 W/cm^2^, respectively.

The distance maintained between the camera and the subject was in accordance with the recommended parameters for the clinical screening of the oral mucosa. The angle of inclination was specifically adjusted to ensure that it did not obstruct or obscure the photographed area, as illustrated in Figure 2a. 

The plates subjected to this illumination method were captured using a camera, specifically the DMC-G80 Lumix Panasonic Corporation camera from Osaka, Japan, equipped with a Lumix Panasonic H-FS 2060, 12-60 micro-HD lens from Tokyo, Japan. A yellow filter with a diameter of 52 mm (Protect-Laserschutz, Nuremberg, Germany) was utilized in the imaging process. This filter was selected for its ability to effectively absorb light in the 160 nm to 450 nm range, which is crucial for exciting autofluorescence, while also offering a strong transmission rate of 76% above this range. This made it ideal for capturing and analyzing autofluorescent emissions in our experiments. 

Photographic documentation was acquired at fixed intervals, specifically after 48, 72, 96, 120, and 144 h of incubation, maintaining consistent camera settings throughout.

### 2.4. Qualitative Visual Assessment of Images

Qualitative visual assessments of images acquired at 48, 72, 96, 120, and 144 h following 405 nm irradiation were performed with the addition of supplemental yellow filtration to determine the presence of red/orange autofluorescence among the different *Candida* strains and colonies.

This analysis followed an initial assessment of the images captured under white light illumination after 48 h of plate incubation. The image analysis was performed independently by a team of 3 researchers, after which a consensus description including several key elements was formulated. The documented assessments included observations of the presence of red/orange autofluorescence (R/OF) in relation to individual strains, colonies, and various observation times. It also encompassed monitoring changes in the intensity of R/OF over the specified observation periods. Furthermore, the assessments provided detailed descriptions of the characteristic patterns of intensity distribution within individual colonies.

Quantitative hue analysis and intra-colony distribution—image processing methodology: Clinical autofluorescence evaluations rely on visual inspection by clinicians. Therefore, a color quantification and mapping approach relevant to human color perception was pursued. Of the existing color systems, including RGB (Red, Green, Blue), HSV/HSB (Hue, Saturation, Value / Hue, Saturation, Brightness), and CIELAB (International Commission on Illumination), the HSV/HSB model was selected for our analysis. The HSV color space is extensively applied in image retrieval contexts, aligns with human visual sensation, and enables the robust characterization of image content [28,29,30,31].

The HSV color space can be visualized as a cylindrical coordinate system featuring three essential variables. The variable H corresponds to hue, symbolizing the perceived colors, namely red, yellow, green, and blue, or various combinations thereof. Saturation (S) denotes the relative purity of a color or the extent to which it is mixed with white, while value (V) quantifies brightness concerning an equivalently illuminated white color [32,33].

**Figure 3:**
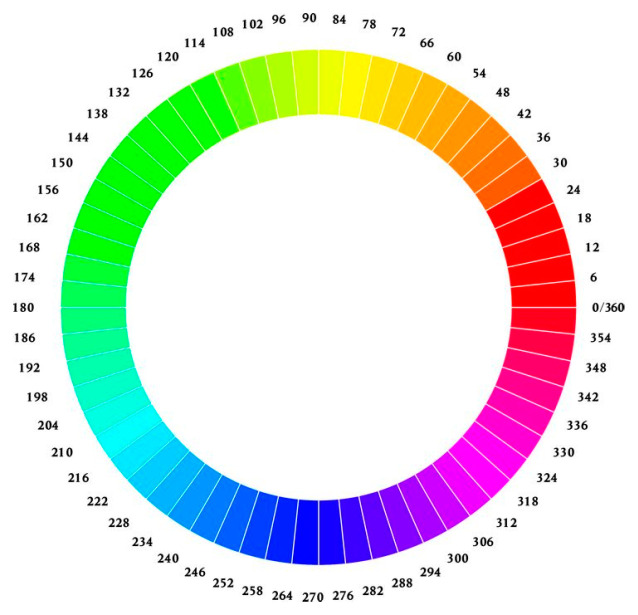
Color wheels showcasing hues arranged radially in a circular fashion.

In this cylindrical coordinate system, H represents the angle of rotation and spans a range from 0 to 360 degrees. S designates the radius’s magnitude, within a range of 0 to 100, signifying the saturation level. V corresponds to the cylinder’s height, which ranges from 0 to 100, denoting the brightness (Figure 3) [33].

Of the HSV variables, hue (H) was determined to be the most informative for characterizing colony color. The minimum hue value (H_min_) of each colony was quantified, with H values of 0–60 corresponding to red and orange spectra (Figure 3). Hue distribution analysis was also conducted along a diameter bisecting the colony center parallel to the image baseline. Image evaluations and analyses were performed using the GNU Image Manipulation Program (GIMP) version 2.99.16 [34] and ImageJ-Fiji version 1.53j (US National Institutes of Health, Bethesda, MD, USA) [35] software platforms. 

### 2.5. Quantification of Colony Minimum and Maximum Hue Values

For each *Candida* strain, at 48 h post-incubation, colony images were isolated from the 405 nm laser irradiation photographs using the GIMP software. A perimeter template was generated for each colony and applied to delineate the same area over subsequent days, normalizing for growth (Figure 1b). This procedure was performed sequentially for all 9 visible colonies in each image and at every observational timepoint. The resulting colony images were analyzed using ImageJ-Fiji to quantify the minimum and maximum hue (H) values within each defined area over time. Equivalent measurements were obtained for the red and orange quantum dot standards (bottom row) and SDA autofluorescence control (green). 

### 2.6. Electronic Analysis of Intra-Colony Hue Distribution

GIMP software was utilized to decompose the colony images into separate hue, saturation, and value grayscale layers. The hue distribution data were then exported. In ImageJ-Fiji, a bisecting diameter segment that spanned the entire colony area parallel to the image baseline and through the vertical center axis was defined for each colony (Figure 1c). The segment was superimposed onto the H-image for each colony within the test strain. Segment placement and dimensions measurements were initially performed on the first image of the test colony, corresponding to the second day of the study. This process was replicated for all colonies. The recorded values associated with the diameter’s dimensions and placement location were compiled in a macro file. Subsequently, the macro file was executed to analyze the images related to each colony whilst preserving the same diameter values throughout subsequent observation days. This systematic approach ensured consistency and accuracy in the hue distribution assessment.

### 2.7. Statistical Analyses

Quantitative data are presented as mean ± standard deviation (SD). Statistical differences among the data sets were assessed using an analysis of variance (ANOVA). Post hoc comparisons were conducted with the Newman–Keuls test to further analyze and compare the results. A *p*-value of ≤0.05 was established as the threshold for indicating statistically significant differences. All statistical analyses were executed utilizing Statistica version 7.1 PL software by StatSoft, located in Krakow, Poland.

## 3. Results

### 3.1. Visual Assessment of Colony Growth and Sample Contamination

White-light imaging conducted at 48 h post-incubation revealed typical intra-strain variation in colony morphology across all *Candida* strains tested. However, no significant inter-strain differences in growth were observed. *C. albicans* ATCC 60193 and *C. krusei* ATCC 14243 displayed the most uniform colony development by day 2. 

**Figure 4:**
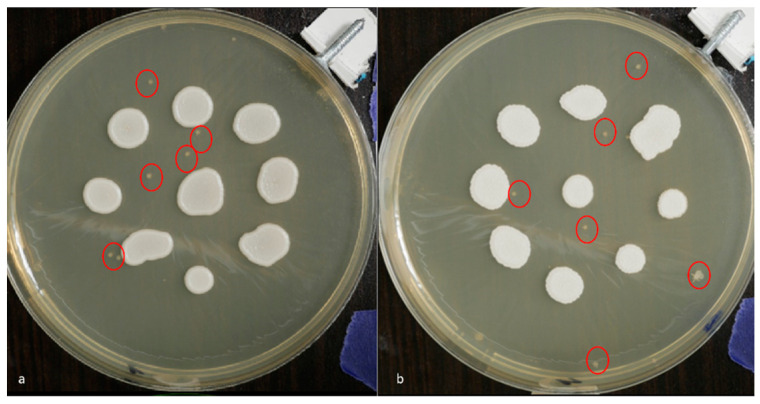
White-light photographic images acquired at 48 h post-inoculation depicting contaminating microbial colonies (indicated by red circles) on plates inoculated with (**a**) *C. albicans* ATCC 60193 and (**b**) *C. krusei* ATCC 14243.

Minor contaminating microorganism colonies were noted on the plates inoculated with *C. albicans* ATCC 10231, *C. krusei* ATCC 14243, and *C. parapsilosis* ATCC 20019 but were considered negligible due to a lack of contact with the *Candida* colonies under study (Figure 4).

### 3.2. Qualitative Assessment of Autofluorescence

**Figure 5:**
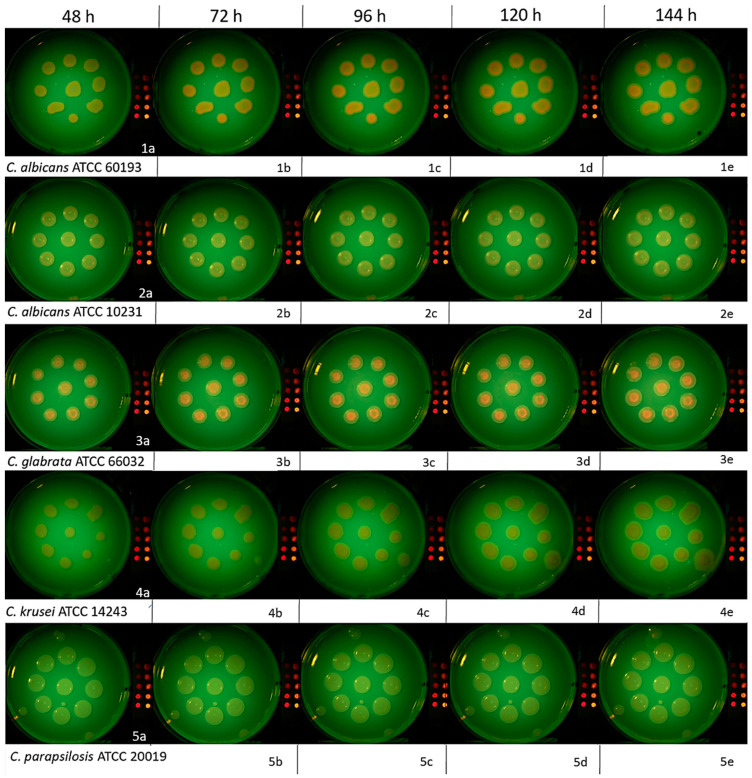
Photographic images of *C. albicans* ATCC 60193, *C. albicans* ATCC 10231, *C. glabrata* ATCC 66032, C. *krusei* ATCC 14243, and *C. parapsilosis* ATCC 20019 colonies on SDA plus 4% glucose at 48, 72, 96, 120, and 144 h post-incubation. Imaging was conducted under 405 nm irradiation with supplemental yellow filtration. The division into subfigures 1a, 2b etc. has been introduced to make it easier to find images described in the text.

At 48 h post-incubation, a subjective visual evaluation of the images acquired under 405 nm illumination with supplemental yellow filtration revealed variable red/orange autofluorescence across the different *Candida* strains. The most pronounced R/OF was observed on the plate containing the *C. albicans* ATCC 60193 strain and *C. glabrata* ATCC 66032. In both cases, this autofluorescence was consistent across all nine colonies. However, its distribution within the colonies exhibited a characteristic pattern with a more intense border rim and a less intense central region (Figure 5(1a,3a)). 

The *C. albicans* ATCC 10231 colonies exhibited similar but less intense R/OF patterns compared to the *C. albicans* ATCC 60193 and *C. glabrata* ATCC 66032 strains (Figure 5(2a)). However, the plate with the *C. krusei* ATCC 14243 strain exhibited a different color and a distinct autofluorescence distribution within the colony. Specifically, it featured a more intense central region and an absence of peripheral autofluorescence. This characteristic was consistent across all nine colonies (Figure 5(4a)). Conversely, no R/OF was detected in any of the colonies of *C. parapsilosis* ATCC 20019 (Figure 5(5a)).

Subsequent observations of the *C. albicans* ATCC 60193 strain after 72 h (Figure 5(1b)), 96 h (Figure 5(1c)), 120 h (Figure 5(1d)), and 144 h (Figure 5(1e)) revealed a noticeable increase in the intensity of red/orange autofluorescence after the third day of observation in all colonies. This heightened intensity was sustained at the same level in the days that followed. The visible red/orange autofluorescence still exhibited the distinctive pattern of a more intense rim and a less intense center. The color remained clear and vivid. Despite daily increases in the colony’s volume, R/OF was consistently present, and its intensity remained unaltered (Figure 5(1b–1e)).

All the observational characteristics described above for the *C. albicans* ATCC 60193 strain were likewise observed and attributed to the *C. glabrata* ATCC 66032 strain (Figure 5(3c–3e)).

Subsequent observations of the *C. albicans* ATCC 10231 strain after 72 h (Figure 5(2b)), 96 h (Figure 5(2c)), 120 h (Figure 5(2d)), and 144 h (Figure 5(2e)) revealed a pattern similar to that of the previously evaluated strain. There was an increase in the intensity of R/OF after 72 h, which persisted at a similar level in the following days of observation. However, it is worth noting that the intensity was not as high as that observed for *C. albicans* ATCC 60193 and *C. glabrata* ATCC 66032. Furthermore, the distribution of intensity within the colony also exhibited the characteristic pattern of a more intense rim and a weaker center. Despite daily increases in the colony’s volume, no red/orange autofluorescence was detected in the new growth sessions, and this pattern remained consistent over time (Figure 5(2b–2e)). 

Observations of the *C. krusei* ATCC 34135 strain after 72 h (Figure 5(4b)), 96 h (Figure 5(4c)), 120 h (Figure 5(4d)), and 144 h (Figure 5(4e)) indicated a gradual increase in autofluorescence intensity in the subsequent days of observation. The colony exhibited relatively homogeneous intensity with a slight rim of no autofluorescence around the periphery of the colony. Notably, the color of the autofluorescence in this strain appeared more subdued and differed in hue from that observed in other strains. While daily increments in colony size did not reveal red/orange autofluorescence, a distinct feature of this strain was that, over time (after 24 h of observation), a color of similar intensity to the older regions began to appear (Figure 5(4b–4e)).

Examinations of *C. parapsilosis* ATCC 20019 at 72, 96, 120, and 144 h revealed a complete absence of R/OF in all nine colonies analyzed at every timepoint assessed (Figure 5(5b–5e)).

### 3.3. Quantitative Colony Hue Analysis—Electronic Evaluation

**Figure 6:**
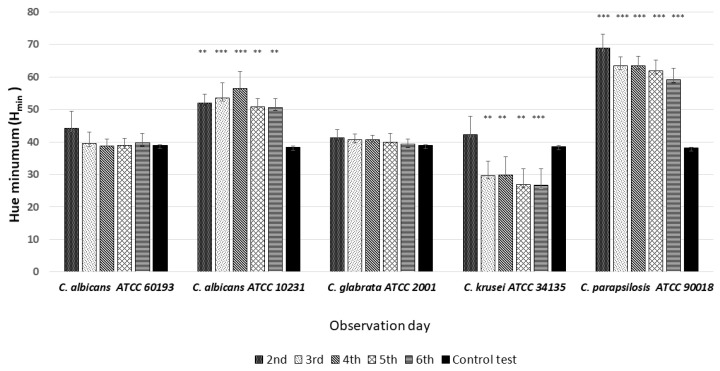
An illustration of the comparison of the minimum hue values (H_min_) for the colonies from each of the five *Candida* strains on the 2nd, 3rd, 4th, 5th, and 6th days of observation. (*** *p* < 0.001; ** *p* < 0.01).

HSV analysis revealed strain-specific differences in minimum hue values (H_min_). Notably, H_min_ values falling within the range of 39–60, corresponding to the orange color (Figure 3 and Figure 6), were consistently observed in strains *C. albicans* ATCC 60193, *C. albicans* ATCC 10231, and *C. glabrata* ATCC 66032 across all planned observation times. Furthermore, when compared to the H_min_ values obtained from the orange dot of the control group (Qdot TM 655), no statistically significant differences were identified in strains *C. albicans* ATCC 60193 and *C. glabrata* ATCC 66032. Although the H_min_ values of strain *C. albicans* ATCC 10231 also fell within the orange color range, only the comparison of this strain with the same control yielded statistically significant results (*p* < 0.001 ***) (Figure 6). 

In the case of strain *C. krusei* ATCC 14243, the H_min_ values consistently ranged from 25 to 30, corresponding to the red color (Figure 3 and Figure 6) (days 3 to 6). These values did exhibit statistically significant differences when compared to the H_min_ obtained from the orange dot control group (Qdot TM 655) (*p* < 0.01 **) (Figure 6).

In strain *C. parapsilosis* ATCC 20019, the H_min_ values, consistently falling within the range of 60–70, extended beyond the orange color range, aligning with the yellow color (Figure 3 and Figure 6). Importantly, these values did exhibit statistically significant differences (*p* < 0.001 ***) when compared to the H_min_ values obtained from the orange dot control group (Qdot TM 655) at all observation times (Figure 6).

**Figure 7:**
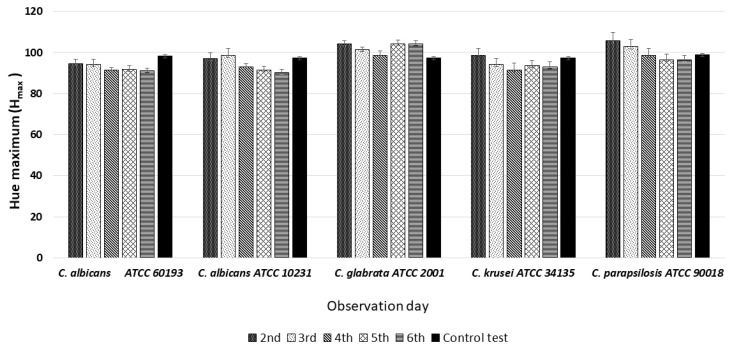
An illustration of the comparison of the maximum hue values (H_max_) for the colonies from each of the five *Candida* strains on the 2nd, 3rd, 4th, 5th, and 6th days of observation.

HSV analysis also revealed slight differences in maximum hue values (H_max_). Notably, the H_max_ values for all observed strains, at all observation times, fell within the range of 90–110, corresponding to the green color (Figure 3 and Figure 7). Furthermore, when compared to the H_max_ values obtained from the green control group, no statistically significant differences were identified between any of the *Candida* strains tested (Figure 7). 

### 3.4. Electronic Analysis of Intra-Colony Hue Distribution

The study involved examining the hue distribution along the diameter of individual colonies, as detailed in the Section 2.6. Specifically, the analysis focused on minimum hue (H_min_) values within three equal segments of the colony diameter (left, middle, and right). The objective of this analysis was to examine the variance in autofluorescence hue between the periphery and center of the colony.

**Figure 8:**
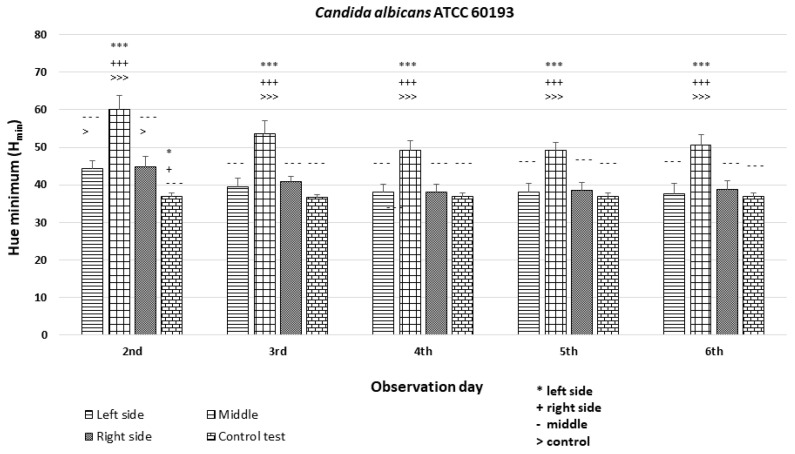
An illustration of the comparison of the minimum hue values (H_min_) for the colonies from the left, middle, and right side of the diameter of each *Candida albicans* ATCC 60193 strain on the 2nd, 3rd, 4th, 5th, and 6th days of observation. (***, +++, ---, >>>—*p* < 0.001; *, +, >—*p* < 0.05).

**Figure 9:**
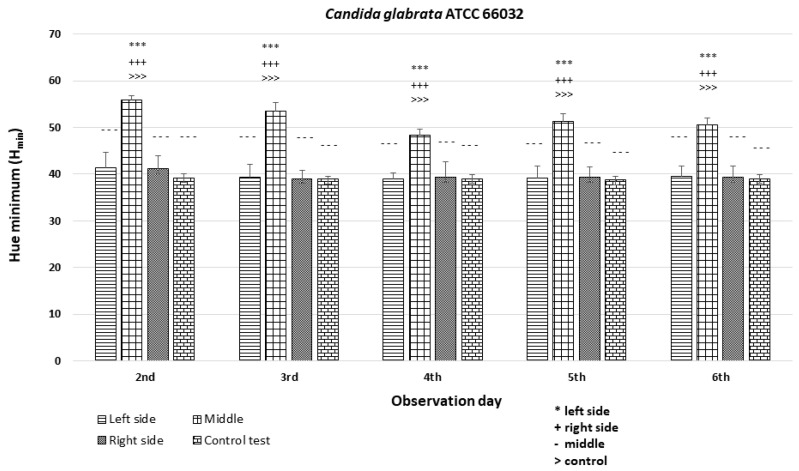
An illustration of the comparison of the minimum hue values (H_min_) for the colonies from the left, middle, and right side of the diameter of *Candida glabrata* ATCC 66032 on the 2nd, 3rd, 4th, 5th, and 6th days of observation. (***, +++, ---, >>>—*p* < 0.001).

The analysis revealed significant differences among the strains and minor differences within the colonies (*p* < 0.001 ***). In the case of strains *C. albicans* ATCC 60193 (Figure 8) and *C. glabrata* ATCC 66032 (Figure 9), the H_min_ values on the left and right sides of the colonies were similar to each other and resembled the H_min_ of the orange dot control. Nevertheless, a statistically significant disparity in fluorescence intensity was observed in the middle section of the diameter compared to both sides and the control. 

**Figure 10:**
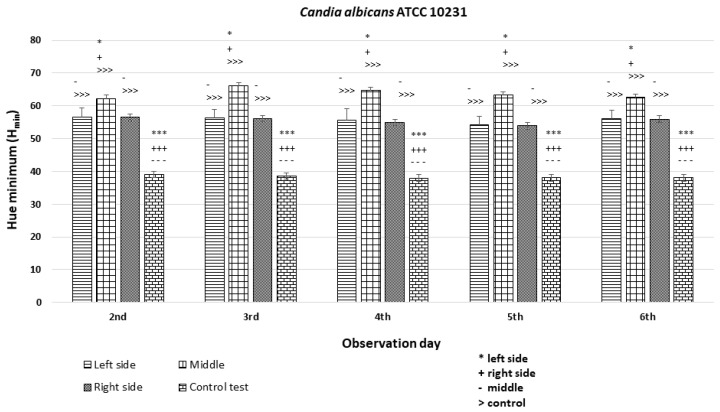
An illustration of the comparison of the minimum hue values (H_min_) for the colonies from the left, middle, and right side of the diameter of *Candida albicans* ATCC 10231 on the 2nd, 3rd, 4th, 5th, and 6th days of observation. (***, +++, ---, >>>—*p* < 0.001; *, +, -—*p* < 0.05).

The *C. albicans* ATCC 10231 strain exhibited H_min_ values on the left and right sides of the colony that were similar to each other and significantly different (*p* < 0.001 ***) from the H_min_ of the orange dot control, although they fell within the range of H = 40–60 corresponding to the orange hue. Statistically significant differences in fluorescence intensity were also observed in the middle section of the diameter compared to both sides and the control (Figure 10). 

**Figure 11:**
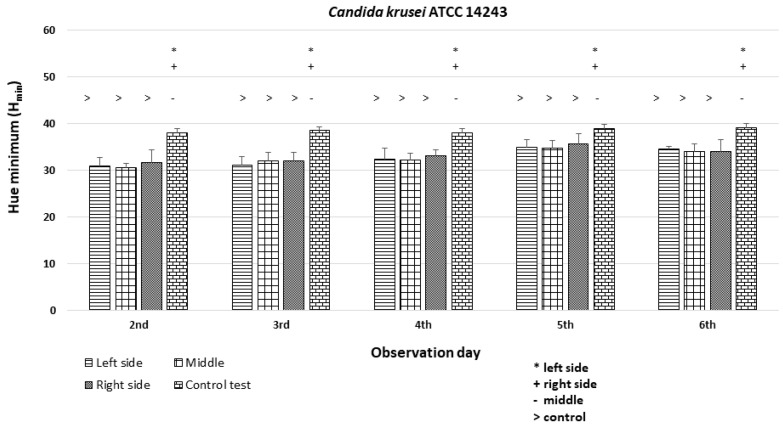
An illustration of the comparison of the minimum hue values (H_min_) for the colonies from the left, middle, and right side of the diameter of *Candida krusei* ATCC 14243 on the 2nd, 3rd, 4th, 5th, and 6th days of observation. (*, +, -, >—*p* < 0.05).

The *C. krusei* ATCC 14243 exhibited H_min_ values that were similar among the left, middle, and right sides of the colonies (H_min_ = 30–35) and significantly different (*p* < 0.05 *) from the H_min_ of the orange dot control (Figure 11). 

**Figure 12:**
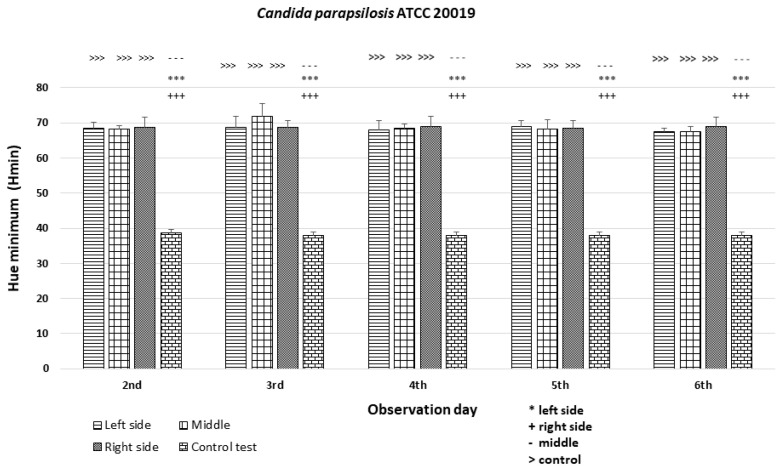
An illustration of the comparison of the minimum hue values (H_min_) for the colonies from the left, middle, and right side of the diameter of *Candida parapsilosis* ATCC 20019 on the 2nd, 3rd, 4th, 5th, and 6th days of observation. (***, +++, ---, >>>—*p* < 0.001).

In the case of the *C. parapsilosis* strain ATCC 20019, the H_min_ values were comparable among the left, middle, and right sides of the colonies (H_min_ = 68–72) and significantly different (*p* < 0.001 ***) from the H_min_ of the orange dot control (Figure 12).

## 4. Discussion

The application of autofluorescence for imaging oral mucosal lesions has a historical context dating back to 1987, when Harris and Werkhaven conducted pioneering research in this field in Chicago. They employed a fluorescent lamp emitting light in the range of 315 to 440 nm with a peak at 370 nm, along with Laser-Guard Argon goggles. Their study, conducted on an animal model revealed the presence of autofluorescence in both tumor and healthy mucosal tissues. Interestingly, the tumor tissues exhibited a distinct red autofluorescence, which was attributed to the presence of endogenous porphyrins [36].

The distinctive bright red/orange fluorescence of malignant tumor tissues was also observed in the 1996, 1999, and 2000 studies by Onizawa et al. [37,38,39]. Their work involved illuminating the oral mucosa with ultraviolet flash lamps emitting light at 360 nm, coupled with the use of a yellow filter. During their studies, a discernible pattern emerged, wherein nearly all malignant lesions exhibited a striking bright red/orange/pink fluorescence. In contrast, only a limited number of benign lesions displayed such fluorescence characteristics [37,38,39].

Subsequent investigations have expanded our understanding of the variations in porphyrin content between healthy and neoplastic tissues. It is now recognized that these distinctions arise not solely from differences in endogenous porphyrin concentrations but also from the presence of porphyrin-producing microorganisms [40]. Bacteria and yeasts are abundant in necrotic and nodular lesions with ulceration, as well as in healthy mucosal areas such as the dorsal surface of the tongue and dental calculus. As a result, the presence of intense red fluorescence in the mucous membrane does not necessarily indicate a neoplastic transformation [40].

The objective of this laboratory experiment was to investigate the presence of red/orange autofluorescence in isolated colonies of different *Candida* strains when exposed to 405 nm laser light. The chosen strains encompass those commonly associated with saprophytic growth and/or those implicated in oral candidiasis. To address this, we included two distinct strains of *C. albicans* and three strains of non-*albicans Candida* which are frequently encountered in patients with intact immune systems alongside cancer [41,42,43].

The results obtained in this study through both visual observations and electronic analysis confirm the presence of red/orange autofluorescence in most of the analyzed *Candida* strains. Nevertheless, it was observed that individual strains exhibit variations in terms of hue and intensity.

Utilizing a laser setup typically used for clinical mucosal lesion diagnosis, a visual assessment was conducted as the initial step, revealing that the colonies emitted non-uniform orange fluorescence with a patchy surface pattern. These findings align with the observations made by Kang et al. in their study involving seven bacterial strains (*Streptococcus mutans*, *Lactobacillus gasseri*, *Enterococcus faecium*, *Fusobacterium nucleatum*, *Porphyromonas gingivalis*, *Veillonella parvula*, and *Actinomyces israelii*) present in the oral cavity. Kang et al. noted significant differences in autofluorescence characteristics under 405 nm light, which they associated with the presence of various intracellular fluorophores in these bacteria [44]. Furthermore, it has been observed that the autofluorescence of individual bacterial strains can change when multiple strains coexist within a biofilm. For instance, the coexistence of *Fusobacterium nucleatum* with *Porphyromonas gingivalis* can lead to alterations in autofluorescence characteristics [45]. Similarly, *Porphyromonas micros* has been reported to exhibit red fluorescence in the proximity of *Porphyromonas gingivalis* [45]. These observations lend support to the quorum sensing (QS) theory within a biofilm. QS involves a cell-to-cell signaling system that relies on signaling molecules secreted by various microbial species, impacting diverse phenotypes such as bioluminescence, virulence, sporulation, adhesion, and biofilm formation [46,47,48].

The electronic HSV color evaluation system was employed to provide an objective assessment of autofluorescence, with a specific focus on the hue value. This system was designed to mimic the way the human eye perceives colors, where all colors are interpreted as light resulting from illumination. The color space description model proposed by Alvey Ray Smith in 1978 served as the basis for this approach [49]. According to this model, colors are derived from white light, with their specific appearance being determined by the absorption and reflection of different parts of the spectrum by illuminated objects. This model finds wide applications across various scientific and technological domains, including medical research. For instance, it has been employed in the analysis of kidney histology images [29], in the study of patients undergoing treatment for ischemic heart disease to assess tongue appearance [30], and as a foundational approach for comprehensive image analysis in medicine [31].

The capability of this system to separate the hue, saturation, and value components offers significant opportunities for precise image analysis. Our analysis validated the visually observed variations in hues and the non-uniform distribution of hues within the colonies.

The results we obtained also indicate the potential for applying digital image processing for the automated assessment of *Candida* spp. This digital approach can substantially expedite the diagnostic process and facilitate the swift evaluation of yeast presence in clinical samples.

We also observed that the color intensity in the early stages of colony maturation (for all strains displaying R/OF) increased and then stabilized at a consistent level. Similar findings were reported in a clinical observational study conducted by van der Veen et al. In this study, among patients who had ceased oral hygiene, faint red autofluorescence became noticeable only on the second day in the majority of study participants, and it grew in intensity over the course of the experiment [22]. The absence or very low presence of R/OF in some individuals towards the end of the study was correlated with a low incidence of periodontitis [22]. A study by Kim et al. also observed a similar relationship. The emission of red fluorescence is associated with the maturation of dental microcosm biofilms, with mature biofilms cultured for at least 3 days emitting red fluorescence [50]. Hence, it was considered that the presence of red/orange autofluorescence on the oral mucosa, such as the specialized membrane of the tongue or in the marginal periodontium, may serve as a valuable non-specific and rapid indicator of the presence of bacteria or yeasts. This could signal the need for the further swab-based identification of the specific strain and assessment of its susceptibility to available antimicrobial treatments.

Rapidly assessing the potential coexistence of microbial superinfections within various pathological lesions on the oral mucosa, particularly in the context of pre-malignant and neoplastic lesions, is of paramount importance. Additional chemical irritation resulting from toxins and enzymes released by microorganisms can alter the appearance of the lesion and contribute to the progression and dissemination of the underlying disease.

The outcomes of studies by various researchers further substantiate the potential applications of assessing red/orange autofluorescence in dentistry. For instance, in a study involving 44 teeth grouped according to the severity of caries, it was found that teeth with caries exhibited red-like autofluorescence when exposed to 405 nm light, with the color intensity being correlated with the severity of caries [51]. Other authors evaluated whether Quantitative Light-induced Fluorescence-Digital (QLF-D) can detect the levels of cariogenicity of dental microcosm biofilms by assessing the red fluorescence intensity after 10 days of incubation in 24-well microplates. They concluded that QLF-D can assess the cariogenic levels of dental plaque based on the intensity of red fluorescence [52]. Furthermore, *Candida* fluorescence is employed in laboratory tests, enabling expedited results. An example of this is the use of a fluorescent laser with calcofluor to detect fungi [53,54,55].

In a scientific study conducted on 120 patients, an investigation involving the visual examination of the tongue and the assessment of red/orange autofluorescence (R/OF) was carried out on the dorsal surface of the tongue using 405 nm light. Simultaneously, swabs were subjected to laboratory examination. This comprehensive analysis unveiled a significant correlation between the presence of *Candida* species and the observed autofluorescence on the upper part of the tongue [13]. However, it is essential to acknowledge that limited knowledge exists regarding the potential impact of external factors on microbial autofluorescence. Factors such as dietary habits and the use of mouthwash may influence autofluorescence, and these variables warrant further investigation. Dietary sugars, particularly glucose, have been shown to influence the pathogenicity of *C. albicans* [56,57]. Jin et al. [56] found that galactose reduced biofilm formation, while glucose promoted stress resistance and gene regulation [57]. The presence of serum and dietary sugars also affected the population and structure of mixed *C. albicans* and *E. coli* biofilms [58]. These findings suggest that dietary factors, including excess sugar, can play a significant role in the pathogenicity of *C. albicans*.

Excess sugar in many patients’ diets appears to significantly influence the red/orange autofluorescence (R/OF) of *Candida* strains [59]. This is particularly relevant in the case of *Candida albicans*, a common human fungal pathogen that can cause a range of infections, from mucosal to life-threatening bloodstream infections [60]. The ability of *C. albicans* to switch between different phenotypic states, including the yeast and mycelial forms, is a key factor in its pathogenicity [61]. The growth of *C. albicans hyphae*, a form associated with disease, is regulated by a network of signal transduction pathways [62].

The pH of the environment can also significantly affect the autofluorescence of *Candida strains*, particularly *Candida albicans*. This is due to the presence of various virulent factors in the cell wall and the ability of the fungus to form biofilms which can be influenced by changes in pH [63,64].

It is worth noting that the autofluorescence of microorganisms can be subject to variation depending on the specific growth medium utilized. Certain studies have employed culture media with added components such as vitamin K, blood, and hemin to facilitate the production of porphyrins by bacteria [19,22,65,66]. Further research is needed to fully understand the mechanisms underlying this phenomenon.

It is important to emphasize that the autofluorescence of *Candida* when exposed to 405 nm light is attributed to porphyrins [13,19]. Porphyrins constitute a diverse group of organic chemical compounds derived from porphine that feature a heterocyclic structure composed of four pyrrole rings. The presence of numerous π electrons within this structure is responsible for generating the red/orange autofluorescence phenomenon [19,20].

The capability for variable autofluorescence among microorganisms is primarily attributed to the differential quantities of the porphyrins present within their cells, which can influence the distinct luminescence observed [45]. Porphyrins are synthesized by microorganisms as part of their biosynthetic processes [44]. Among the well-known endogenous porphyrins occurring in cell structures are protporphyrin IX, coproporphyrin III (a protoporphyrin IX precursor), and uroporphyrin [67,68]. Each of these compounds exhibits unique luminescence properties, with peak intensities spanning the range of 607–623 nm [25,51], 635 nm [69], and 683 nm [17].

Endogenous porphyrins are typically distributed across various morphological regions of the cell, predominantly found within the cytoplasm of microbial cells and their cell membranes, encompassing both bacteria and *Candida* [5]. A study conducted by Plavskii et al. employed fluorescence spectrometry to analyze extracts from *Candida albicans* cells. This analysis was performed by adding the non-ionic detergent Triton X-100 to the cells. The results of the study confirmed the presence of metal-free porphyrins, including coproporphyrin and uroporphyrin, whilst protoporphyrin was not detected in this investigation [16].

The increasing knowledge of the biochemical aspects of *Candida* R/OF has paved the way for assessing the presence of yeasts and other microorganisms not only in the oral mucosa but also in various medical fields, including dermatology [70,71,72].

### Limitations and Future Research

To fully harness the diagnostic potential of autofluorescence, further research involving patient groups is essential. Comparative studies with other available diagnostic techniques are necessary to evaluate the efficacy and reliability of this method. Additionally, exploring the utilization of different wavelengths of light for autofluorescence testing may offer further diagnostic insights. A limitation of this study is the fact that we only used reference strains and monocellular biofilms, but this was vital for a precise assessment of the individual R/OF capacity of each of the tested strains. The upcoming phase of our research will focus on evaluating the fluorescence of *Candida* strains and other microbes from patient swabs diagnosed with oral mucosal candidiasis studied in both single-species and, ideally, in multi-species biofilms. Another limitation was the lack of R/OF assessment at the single-cell level, allowing for the evaluation of the distribution of porphyrins within the cell’s organelles. This will also be incorporated into our next study. This study marks a significant stride toward the development of efficient, rapid, and non-invasive diagnostic tools in dentistry, medicine, and diagnostic and laboratory applications.

## 5. Conclusions

This study demonstrates that most of the *Candida* strains tested exhibit red/orange autofluorescence under 405 nm laser light, with variations in hue and intensity across strains. Visual and electronic analyses confirmed these findings. These results, particularly the persistent R/OF intensity and distinctive intra-colony patterns, contribute to our understanding of *Candida’s* autofluorescence properties and its clinical significance, particularly for diagnosing oral candidiasis and potentially other related conditions, suggesting its potential as a rapid, non-invasive detection method for *Candida* in clinical settings.

## 6. Clinical Significance

The use of 405 nm laser light enables a rapid, non-invasive assessment of *Candida* spp. on both healthy and diseased mucosa, detecting R/OF even in challenging areas of the oral cavity. The observed autofluorescence of *Candida* strains under specific light conditions can aid in the rapid identification of these pathogens, streamlining the detection and diagnostic process in oral health screenings and opening up new possibilities for integrating R/OF detection into routine dental screenings, thereby enhancing both preventive care and treatment strategies and potentially improving the management of candidiasis and other *Candid*-related pathologies. This method could form the basis for specialized tests to facilitate effective treatment. Additionally, our findings open avenues for employing existing 405 nm light-based devices to detect and diagnose pre-cancerous and cancerous lesions, marking a novel clinical application of this technology.

## Data Availability

The authors will make the data of this study available upon request.

## References

[1] Mbakwem-Aniebo C., Osadebe A.U., Athanasonny E., Okonko I.O. (2020). Prevalence of Candida spp. and Age-Related Disparities Amongst Women Presenting with Vaginitis at the Obstetrics and Gynaecology (O&G) Clinic in a Tertiary Hospital in Port Harcourt, Nigeria. Afr. Health Sci..

[2] Zafar S., Fatima K., Faryal R. (2020). Prevalence of *Virulent candida* spp. in Complicated Urinary Tract Infection of Nephrolithiatic Patients from Surgical Units of Tertiary Care Hospitals Islamabad. J. Mycol. Med..

[3] Wiench R., Skaba D., Matys J., Grzech-Leśniak K. (2021). Efficacy of Toluidine Blue-Mediated Antimicrobial Photodynamic Therapy on Candida spp. A Systematic Review. Antibiotics.

[4] Beighton D., Lynch E. (1993). Relationships between Yeasts and Primary Root-Caries Lesions. Gerodontology.

[5] Pappas P.G., Lionakis M.S., Arendrup M.C., Ostrosky-Zeichner L., Kullberg B.J. (2018). Invasive Candidiasis. Nat. Rev. Dis. Primers.

[6] Segrelles-Calvo G., de SAraújo G.R., Llopis-Pastor E., Carrillo J., Hernández-Hernández M., Rey L., Melean N.R., Escribano I., Antón E., Zamarro C. (2021). *Candida* spp. co-infection in COVID-19 patients with severe pneumonia: Prevalence study and associated risk factors. Respir. Med..

[7] Freitas V.A.Q., Santos A.S., Zara A.L.S.A., Costa C.R., Godoy C.S.M., Soares R.B.A., Ataídes F.S., Silva M.D.R.R. (2023). Distribution and antifungal susceptibility profiles of Candida species isolated from people living with HIV/AIDS in a public hospital in Goiânia, GO, Brazil. Braz. J. Microbiol..

[8] Akpan A., Morgan R. (2002). Oral Candidiasis. Postgrad. Med. J..

[9] Tasso C.O., Ferrisse T.M., de Oliveira A.B., Ribas B.R., Jorge J.H. (2023). Candida Species as Potential Risk Factors for Oral Squamous Cell Carcinoma: Systematic Review and Meta-Analysis. Cancer Epidemiol..

[10] Silva S., Negri M., Henriques M., Oliveira R., Williams D.W., Azeredo J. (2012). Candida glabrata, Candida parapsilosis and Candida tropicalis: Biology, Epidemiology, Pathogenicity and Antifungal Resistance. FEMS Microbiol. Rev..

[11] Graus M.S., Neumann A.K., Timlin J.A. (2017). Hyperspectral Fluorescence Microscopy Detects Autofluorescent Factors That Can Be Exploited as a Diagnostic Method for Candida Species Differentiation. J. Biomed. Opt..

[12] Kobayashi T., Saito T., Ohtani H. (2001). Real-Time Spectroscopy of Transition States in Bacteriorhodopsin During Retinal Isomerization. Nature.

[13] Petruzzi M., Della Vella F., Cassandro A., Mosca A., Di Comite M., Contaldo M., Grassi F.R., Lauritano D. (2019). Dorsal Tongue Porphyrin Autofluorescence and Candida Saprophytism: A Prospective Observational Study. PLoS ONE.

[14] Lane P.M., Gilhuly T., Whitehead P., Zeng H., Poh C.F., Ng S., Williams P.M., Zhang L., Rosin M.P., MacAulay C.E. (2006). Simple Device for the Direct Visualization of Oral-Cavity Tissue Fluorescence. J. Biomed. Opt..

[15] Na R., Stender I.M., Henriksen M., Wulf H.C. (2001). Autofluorescence of Human Skin is Age-Related After Correction for Skin Pigmentation and Redness. J. Investig. Dermatol..

[16] Plavskii V.Y., Mikulich A.V., Tretyakova A.I., Leusenka I.A., Plavskaya L.G., Kazyuchits O.A., Dobysh I.I., Krasnenkova T.P. (2018). Porphyrins and Flavins as Endogenous Acceptors of Optical Radiation of Blue Spectral Region Determining Photoinactivation of Microbial Cells. J. Photochem. Photobiol. B.

[17] De Veld D.C.G., Witjes M.J.H., Sterenborg H.J.C.M., Roodenburg J.L.N. (2005). The Status of In Vivo Autofluorescence Spectroscopy and Imaging for Oral Oncology. Oral Oncol..

[18] McAlpine J.N., El Hallani S., Lam S.F., Kalloger S.E., Luk M., Huntsman D.G., MacAulay C., Gilks C.B., Miller D.M., Lane P.M. (2011). Autofluorescence Imaging Can Identify Preinvasive or Clinically Occult Lesions in Fallopian Tube Epithelium: A Promising Step Towards Screening and Early Detection. Gynecol. Oncol..

[19] Volgenant C.M., van der Veen M.H., de Soet J.J., ten Cate J.M. (2013). Effect of Metalloporphyrins on Red Autofluorescence from Oral Bacteria. Eur. J. Oral Sci..

[20] Volgenant C.M., Hoogenkamp M.A., Krom B.P., Janus M.M., Ten Cate J.M., de Soet J.J., Crielaard W., van der Veen M.H. (2016). Red and Green Fluorescence from Oral Biofilms. PLoS ONE.

[21] de Veld D.C., Skurichina M., Witjes M.J., Duin R.P.W., Sterenborg D.J.C.M., Star W.M., Roodenburg J.L.N. (2003). Autofluorescence Characteristics of Healthy Oral Mucosa at Different Anatomical Sites. Lasers Surg. Med..

[22] van der Veen M.H., Volgenant C.M.C., Keijser B., Jacob Bob M., Crielaard W. (2016). Dynamics of Red Fluorescent Dental Plaque during Experimental Gingivitis—A Cohort Study. J. Dent..

[23] Oriel S., Nitzan Y. (2010). Photoinactivation of Candida albicans by Its Own Endogenous Porphyrins. Curr. Microbiol..

[24] Fraikin Y., Strakhovskaya M.G., Rubin A.B. (1996). The Role of Membrane-Bound Porphyrin-Type Compound as Endogenous Sensitizer in Photodynamic Damage to Yeast Plasma Membranes. J. Photochem. Photobiol. B.

[25] Zhang Y., Zhu Y., Chen J., Wang Y., Sherwood M.E., Murray C.K., Vrahas M.S., Hooper D.C., Hamblin M.R., Dai T. (2016). Antimicrobial Blue Light Inactivation of Candida albicans: In Vitro and In Vivo Studies. Virulence.

[26] Murdoch L.E., McKenzie K., Maclean M., Macgregor S.J., Anderson J.G. (2013). Lethal Effects of High-Intensity Violet 405-nm Light on Saccharomyces cerevisiae, Candida albicans, and on Dormant and Germinating Spores of Aspergillus niger. Fungal Biol..

[27] Hu L., He C., Zhao C., Chen X., Hua H., Yan Z. (2019). Characterization of Oral Candidiasis and the Candida Species Profile in Patients with Oral Mucosal Diseases. Microb. Pathog..

[28] Vadivel A., Sural S., Majumdar A.K. (2005). Human Color Perception in the HSV Space and Its Application in Histogram Generation for Image Retrieval. Color Imaging X Process. Hardcopy Appl..

[29] Kurniastuti I., Yuliati E.N.I., Yudianto F., Wulan T.D. (2022). Determination of Hue Saturation Value (HSV) Color Feature in Kidney Histology Image. J. Phys. Conf. Ser..

[30] Xia Y.-M., Wang Q.-S., Feng X., Xiao X.-A., Wang Y.-Q., Xu Z.-X. (2022). Objective Tongue Diagnosis Based on HSV Color Space: Controlled Study of Tongue Appearance in Patients Treated with Percutaneous Coronary Intervention for Coronary Heart Disease. Intell. Med..

[31] Satrya G.B., Ramatryana I.N.A., Shin S.Y. (2023). Compressive Sensing of Medical Images Based on HSV Color Space. Sensors.

[32] Liu G.-H., Wei Z. (2020). Image Retrieval Using the Fused Perceptual Color Histogram. Comput. Intell. Neurosci..

[33] Zhong H., Wang R. (2022). A Visual-Degradation-Inspired Model with HSV Color-Encoding for Contour Detection. J. Neurosci. Methods.

[34] Kimball S., Mattis P., GIMP Team GNU Image Manipulation Program. 1995–2023. https://www.gimp.org/.

[35] Rasband W.S. (1997–2018). ImageJ.

[36] Harris D.M., Werkhaven J. (1987). Endogenous Porphyrin Fluorescence in Tumors. Lasers Surg. Med..

[37] Onizawa K., Saginoya H., Furuya Y., Yoshida H. (1996). Fluorescence Photography as a Diagnostic Method for Oral Cancer. Cancer Lett..

[38] Onizawa K., Saginoya H., Furuya Y., Yoshida H., Fukuda H. (1999). Usefulness of Fluorescence Photography for Diagnosis of Oral Cancer. Int. J. Oral Maxillofac. Surg..

[39] Onizawa K., Yoshida H., Saginoya H. (2000). Chromatic Analysis of Autofluorescence Emitted from Squamous Cell Carcinomas Arising in the Oral Cavity: A Preliminary Study. Int. J. Oral Maxillofac. Surg..

[40] Betz C.S., Mehlmann M., Rick K., Stepp H., Grevers G., Baumgartner R., and Leunig A. (1999). Autofluorescence Imaging and Spectroscopy of Normal and Malignant Mucosa in Patients with Head and Neck Cancer. Lasers Surg. Med..

[41] Tang B.H.E., Bay J.W., Yeong F.M., Samuel M. (2023). Efficacy and Safety of Echinocandin Monotherapy and Combination Therapy for Immunocompromised Patients with Systemic Candidiasis: A Systematic Review and Meta-Analysis. J. Mycol. Med..

[42] Orlandini R.K., Bepu D.A.N., Saraiva M.D.C.P., Bollela V.R., Motta A.C.F., Lourenço A.G. (2020). Are Candida albicans Isolates from the Oral Cavity of HIV-Infected Patients More Virulent than from Non-HIV-Infected Patients? Systematic Review and Meta-Analysis. Microb. Pathog..

[43] Lorenzo-Pouso A.I., Pérez-Jardón A., Caponio V.C.A., Spirito F., Chamorro-Petronacci C.M., Álvarez-Calderón-Iglesias Ó., Gándara-Vila P., Lo Muzio L., Pérez-Sayáns M. (2022). Oral Chronic Hyperplastic Candidiasis and Its Potential Risk of Malignant Transformation: A Systematic Review and Prevalence Meta-Analysis. J. Fungi.

[44] Kang S.M., de Josselin de Jong E., Higham S.M., Hope C.K., Kim B.I. (2020). Fluorescence Fingerprints of Oral Bacteria. J. Biophotonics.

[45] Lee M.A., Kang S.M., Kim S.Y., Kim J.S., Kim J.B., Jeong S.H. (2018). Fluorescence Change of Fusobacterium nucleatum due to Porphyromonas gingivalis. J. Microbiol..

[46] van der Veen M.H., Thomas R.Z., Huysmans M.C., de Soet J.J. (2006). Red Autofluorescence of Dental Plaque Bacteria. Caries Res..

[47] Taga M.E., Bassler B.L. (2003). Chemical Communication Among Bacteria. Proc. Natl. Acad. Sci. USA.

[48] Jayaraman A., Wood T.K. (2008). Bacterial Quorum Sensing: Signals, Circuits, and Implications for Biofilms and Disease. Annu. Rev. Biomed. Eng..

[49] Smith A.R. (1978). Color Gamut Transform Pairs. Comput. Graph..

[50] Kim Y.S., Lee E.S., Kwon H.K., Kim B.I. (2014). Monitoring the Maturation Process of a Dental Microcosm Biofilm Using the Quantitative Light-induced Fluorescence-Digital (QLF-D). J. Dent..

[51] Chen Q., Zhu H., Xu Y., Lin B., Chen H. (2015). Discrimination of Dental Caries Using Colorimetric Characteristics of Fluorescence Spectrum. Caries Res..

[52] Lee E.S., Kang S.M., Ko H.Y., Kwon H.K., Kim B.I. (2013). Association between the Cariogenicity of a Dental Microcosm Biofilm and Its Red Fluorescence Detected by Quantitative Light-induced Fluorescence-Digital (QLF-D). J. Dent..

[53] Kumaraswamy Naik L.R., Shetty P., Krishna Prasad M.S., Karnaker V.K., Shroff S.E., Madathil L.P. (2016). Fluorescence of Candida in Diagnosis of Oral Candidiasis. Indian J. Dent. Res..

[54] Chinnasamy A., Ramalingam K., Nayak S., Rai V., Gopinath V., Chawla G. (2022). Diagnostic Yield of Calcofluor White in the Identification of Candida albicans in Oral Squamous Cell Carcinoma. J. Oral Maxillofac. Pathol..

[55] Bhavasar R.S., Goje S.K., Takalkar A.A., Ganvir S.M., Hazarey V.K., Gosavi S.R. (2010). Detection of Candida by Calcofluor White. Acta Cytol..

[56] Jin Y., Yip H.K., Samaranayake Y.H., Yau J.Y., Samaranayake L.P. (2003). Biofilm-Forming Ability of Candida albicans is Unlikely to Contribute to High Levels of Oral Yeast Carriage in Cases of Human Immunodeficiency Virus Infection. J. Clin. Microbiol..

[57] Rodaki A., Bohovych I.M., Enjalbert B., Young T., Odds F.C., Gow N.A.R., Brown A.J.P. (2009). Glucose Promotes Stress Resistance in the Fungal Pathogen Candida albicans. Mol. Biol. Cell.

[58] Thein Z.M., Seneviratne C.J., Samaranayake Y.H., Samaranayake L.P. (2009). Community Lifestyle of Candida in Mixed Biofilms: A Mini Review. Mycoses.

[59] Calderone R.A., Fonzi W.A. (2001). Virulence Factors of Candida albicans. Trends Microbiol..

[60] Kim J., Sudbery P.E. (2011). Candida albicans, A Major Human Fungal Pathogen. J. Microbiol..

[61] Biswas S., Van Dijck P., Datta A. (2007). Environmental Sensing and Signal Transduction Pathways Regulating Morpho-pathogenic Determinants of Candida albicans. Microbiol. Mol. Biol. Rev..

[62] Sudbery P.E. (2011). Growth of Candida albicans Hyphae. Nat. Rev. Microbiol..

[63] Chaffin W.L. (2008). Candida albicans Cell Wall Proteins. Microbiol. Mol. Biol. Rev..

[64] Gulati M., Nobile C.J. (2016). Candida albicans Biofilms: Development, Regulation, and Molecular Mechanisms. Microbes Infect..

[65] Lennon A.M., Buchalla W., Brune L., Zimmermann O., Gross U., Attin T. (2006). The Ability of Selected Oral Microorganisms to Emit Red Fluorescence. Caries Res..

[66] Lennon Á.M., Brune L., Techert S., Buchalla W. (2023). Fluorescence Spectroscopy Shows Porphyrins Produced by Cultured Oral Bacteria Differ Depending on Composition of Growth Media. Caries Res..

[67] Shu M., Kuo S., Wang Y., Jiang Y., Liu Y.T., Gallo R.L., Huang C.M. (2013). Porphyrin Metabolisms in Human Skin Commensal Propionibacterium acnes Bacteria: Potential Application to Monitor Human Radiation Risk. Curr. Med. Chem..

[68] Barnard E., Johnson T., Ngo T., Arora U., Leuterio G., McDowell A., and Li H. (2020). Porphyrin Production and Regulation in Cutaneous Propionibacteria. mSphere.

[69] Hope C.K., Strother M., Creber H.K., Higham S.M. (2016). Lethal Photosensitisation of Prevotellaceae under Anaerobic Conditions by Their Endogenous Porphyrins. Photodiagnosis Photodyn. Ther..

[70] Rennie M.Y., Lindvere-Teene L., Tapang K., Linden R. (2017). Point-of-Care Fluorescence Imaging Predicts the Presence of Pathogenic Bacteria in Wounds: A Clinical Study. J. Wound Care.

[71] Jones L.M., Dunham D., Rennie M.Y., Kirman J., Lopez A.J., Keim K.C., Little W., Gomez A., Bourke J., Ng H. (2020). In vitro detection of porphyrin-producing wound bacteria with real-time fluores-cence imaging. Future Microbiol..

[72] Rennie M.Y., Dunham D., Lindvere-Teene L., Raizman R., Hill R., Linden R. (2019). Understanding Real-Time Fluorescence Signals from Bacteria and Wound Tissues Observed with the MolecuLight i:X™. Diagnostics.

